# Effect of Postal-Mailed Nicotine Patches on Tobacco Cessation Among Smokers in Rural Canada: A Randomized Clinical Trial

**DOI:** 10.1001/jamanetworkopen.2023.25206

**Published:** 2023-07-24

**Authors:** John A. Cunningham, Michael Chaiton, Scott T. Leatherdale, Alexandra Godinho, Christina Schell

**Affiliations:** 1 National Addiction Centre, Institute of Psychiatry, Psychology and Neuroscience, Kings College London, London, United Kingdom; 2 Institute for Mental Health Policy Research, Centre for Addiction and Mental Health, Toronto, Ontario, Canada; 3 Department of Psychiatry, University of Toronto, Toronto, Ontario, Canada; 4 Dalla Lana School of Public Health, University of Toronto, Toronto, Ontario, Canada; 5 School of Public Health Sciences, University of Waterloo, Waterloo, Ontario, Canada; 6 Humber River Health Research Institute, Humber River Hospital, Toronto, Ontario, Canada

## Abstract

This randomized clinical trial assesses the efficacy of mailed nicotine patches on cessation of tobacco smoking among adults in rural Canada.

## Introduction

Nicotine replacement therapy (NRT) has been found to promote tobacco cessation in multiple trials.^1,2^ A secondary analysis^3^ of a trial^4^ examining the efficacy of mailed nicotine patches with tobacco cessation indicated that the intervention might have a larger impact in rural regions than in urban areas (odds ratio [OR], 9.59 vs 2.16). The current randomized clinical trial recruited participants from rural regions with the hypothesis that those receiving the NRT package would display substantially greater quit rates (ie, 30-day abstinence) at 6-month follow-up compared with those not offered the package.

## Methods

This study was approved by the standing institutional review board of the Centre for Addictions and Mental Health and followed the (CONSORT) reporting guideline. The trial protocol has been published elsewhere^5^ and is shown in Supplement 1. Informed consent was obtained verbally via telephone, and participants were compensated CAD $20 per survey. Telephone numbers identified as being from rural regions of Canada were called at random. Potential participants were identified from household members by asking for the cigarette smoker (smoking ≥10 cigarettes per day) with the next birthday, who was aged 18 years or older, and who was willing to take part in a baseline and a 6-month follow-up survey asking about their experiences with smoking. Participants who stated that they were interested in receiving free nicotine patches (among questions about aids to stop smoking), who stated that they would use the patches within a week of receiving them at their home to attempt to quit smoking, and who had no health contraindications against using nicotine patches, were randomized to 1 of 2 conditions: (1) told that we had 5-week supplies of nicotine patches and asked if they were willing to have them mailed to their home; and (2) not told anything about receiving nicotine patches. Participants in the intervention condition who agreed to be mailed nicotine patches were sent the patches along with instructions on their use. Participants randomized to the control condition were recontacted after 6 months to take part in another survey. The primary outcome analysis used a logistic regression to examine self-reported 30-day abstinence at 6-month follow-up using an intent-to-treat approach (participants lost to follow-up were assumed to still be smoking). Analyses were conducted on March 27, 2023, using SPSS statistical software version 28 (IBM).

## Results

A total of 1255 participants completed the baseline survey, and 498 participants (mean [SD] age, 56.7 [13.4] years; 244 women [49.0%]) were randomized to the intervention or control condition (Figure). The follow-up rate was 211 participants (85.8%) in the control condition and 212 participants (84.1%) in the intervention condition. The Table provides the baseline demographic and smoking characteristics of the cohort.

**Figure.**
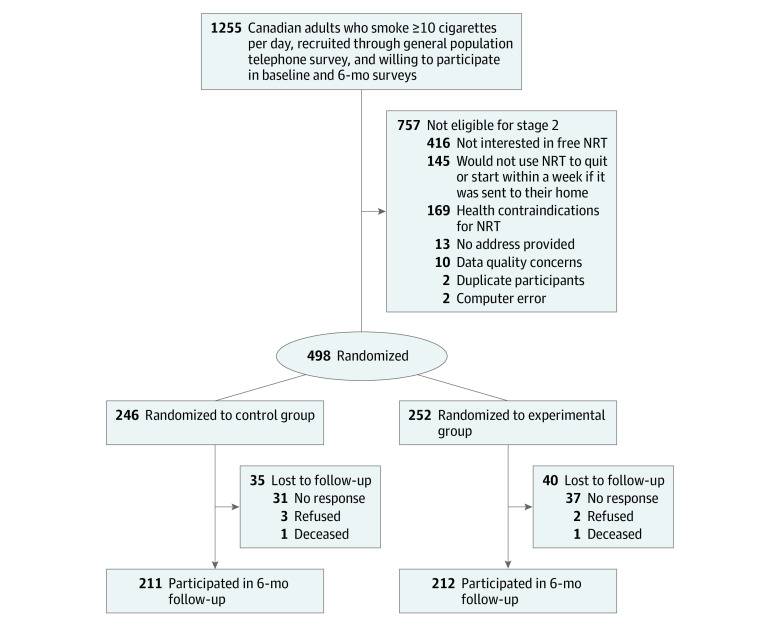
CONSORT Flowchart NRT indicates nicotine replacement therapy.

**Table.** Baseline Demographic and Smoking Characteristics of Participants

Demographic characteristics	Participants, No. (%) (N = 498)	
	Intervention (n = 252)	Control (n = 246)
Age, mean (SD), y	57.4 (12.9)	56.1 (14.0)
Gender		
Female	121 (48.0)	123 (50.0)
Male	130 (51.8)	121 (49.2)
Other	0	2 (0.8)
Missing	1 (<0.1)	0
Married or common-law marriage	129 (51.2)	136 (55.3)
Employed full-time or part-time	134 (53.2)	116 (47.3)
Education level		
Less than high school diploma	77 (30.6)	70 (28.5)
High school diploma	94 (37.3)	92 (37.4)
Any postsecondary	81 (32.1)	84 (34.1)
Household income less than $60 000/y	169 (67.1)	150 (66.7)
Smoking characteristics		
Cigarettes/d, mean (SD), No.	18.7 (9.9)	17.8 (7.5)
Daily smoking, mean (SD), y	31.4 (16.8)	29.5 (16.7)

Participants in the intervention condition were more likely to report 30-day point prevalence abstinence compared with participants in the control condition (28 participants [11.1%] vs 9 participants [3.7%]; OR, 3.29; 95% CI, 1.52-7.13). A complete case analysis of the 423 participants who were successfully followed-up revealed similar findings (28 intervention participants [13.2%] vs 9 control participants [4.3%]; OR, 3.42; 95% CI, 1.57-7.43). Finally, further examination of the postal addresses of participants revealed that a small proportion (71 participants [14.3%]) did not provide a rural address. With these participants removed, the intent-to-treat analysis was repeated and displayed a similar pattern of results (22 intervention participants [10.2%] vs 8 control participants [3.8%]; OR, 2.88; 95% CI, 1.25-6.62).

## Discussion

This randomized clinical trial found further evidence supporting the efficacy of the mailed nicotine patch approach to promote smoking cessation in rural locations where recipients do not have easy access to health services.^6^ A limitation of the trial was a lack of biochemical validation of smoking status.

## Notes

Supplement 1.
Trial Protocol

Supplement 2.
Data Sharing Statement
