# Supplementary material for: Effect of Postal-Mailed Nicotine Patches on Tobacco Cessation Among Smokers in Rural Canada: A Randomized Clinical Trial
Source: JAMA Netw Open. 2023 Jul 24;6(7):e2325206. doi: 10.1001/jamanetworkopen.2023.25206 (PMC10366697; doi:10.1001/jamanetworkopen.2023.25206)
Supplement: Supplement 1. — Trial Protocol [file jamanetwopen-e2325206-s001.pdf]

Trial Protocol
Scientific Abstract

Quitting smoking is the most effective way of reducing the risk of cancer. One way of helping people stop smoking is to provide them with free Nicotine Replacement Therapy (NRT; e.g., nicotine patch), such as when NRT is sent to people by postal mail as part of a mass distribution initiative. Our previous research indicated that the impact of the mailed NRT intervention on increasing quit rates in rural areas may be substantial. The current research project seeks to confirm this finding and to understand the social determinants of health driving these anticipated large effects. This information is essential in order to target limited health resources to regions that are most in need, and who are likely to experience the greatest benefit.

Telephone numbers will be randomly selected from across rural regions of Canada in order to recruit adult smokers interested in completing a smoking survey and willing to be interviewed again in 6 months. The survey will ask participants about their smoking history, demographic characteristics, and a hypothetical question: would they be interested in receiving the nicotine patch if this were provided to them free of charge? Half of the smokers interested in receiving nicotine patches will be selected by chance and offered the NRT package. The other half of smokers will not be offered the nicotine patches. In addition, the municipality where each participant lives will be identified and participant data will be linked to relevant municipal characteristics (e.g., smoking rates, availability of health services). Characteristics of the participants and the municipalities in which they live will be used to explain why the nicotine patch intervention has a larger impact in some rural regions compared to others.

The findings from the proposed RCT are timely and of high relevance as the distribution of nicotine patches has substantial potential to combat the public health problem of cigarette related cancer, other diseases, and premature death from tobacco use. Targeting such tobacco cessation initiatives to rural regions may substantially increase the impact and cost-effectiveness of this intervention, helping to optimize the use of limited prevention resources while aiming to save the maximum number of lives.

Background
In Canada, smoking tobacco accounts for approximately 30% of all cancer deaths and is a risk factor for at least 18 types of cancer.[1,2] Smoking is also a significant negative contributor to numerous other health conditions.[3] Rates of smoking appear to have recently increased in Canada (15% in 2017 from 13% in 2015).[4] Cessation plays an impactful role in the prevention of cancer, where 10 years after quitting the mortality rate from lung cancer is about half of that of a continuing smoker.[5]

Rates of smoking are higher in rural than in urban regions of Canada: Over 6.3 million people live in rural regions of Canada and smoking prevalence is higher in rural compared to urban regions.[6-8] Also crucial – tobacco cessation initiatives have been less successful outside of urban areas and provision of health care is challenging in areas of lower population density.[9,10] Given that Canadian mortality rates are higher in rural than in urban areas,[11] and that a significant component of this difference in mortality rates is due to modifiable risk factors, such as smoking, interventions targeting smoking in these regions could reduce these mortality inequalities.[8,12,13] To reduce the health disparities associated with living in rural areas, new ways of promoting tobacco cessation are needed that are amenable to delivery over large geographic areas. The provision of free nicotine patches sent by postal mail is one such promising option.

Effectiveness of NRT: There has been extensive research evaluating the efficacy of NRT as a means to promote smoking cessation. A Cochrane review of 150 randomized trials involving NRT concluded that NRTs increase the rate of quitting smoking by 50 to 70%, irrespective of the clinical setting in which the smoker is treated.[14,15]

Evidence for the benefits of providing free NRT by postal mail: There are mass distribution initiatives of free NRT ongoing in several countries, including the US[16-18] and Canada (Ontario).[19,20] Further, there is evidence that the distribution of free NRT is **cost-effective**, with the cost of $179 per participant in the Canadian mass distribution initiative.[20] Moreover, a promising aspect about mass distribution of NRT initiatives is that, while it is available to everyone, **vulnerable populations (whether due to mobility issues, or co-existing mental health concerns) are by far the most likely to respond to the initiative and request free NRT**.[20] An additional point as an argument of the benefit of providing free NRT is that it removes a financial barrier to access of NRT for people with low socioeconomic status,[21] a factor that often co-occurs with areas of high smoking rates.[22] Thus, the proposed tobacco cessation initiative is an excellent means of targeting vulnerable populations.

Targeting NRT distribution to regions that may benefit most: Results from our mailed nicotine patch trial indicated that the impact of free NRT distribution may be unexpectedly large in rural areas (OR = 9.59).[7] This effect size is substantial in comparison to that observed in our earlier work on NRT distribution that largely comprised participants from urban/suburban regions (OR = 2.65).[23] While encouraging, this large effect size requires replication to increase confidence in the results because the sample size of participants from rural regions in our earlier trial was small and this was a secondary analysis.

What other factors might predict a large impact of the NRT intervention?: Understanding the regional factors driving the impact of the NRT intervention are essential as it points to a means of targeting limited public health resources to substantially increase the number of people quitting smoking. There is a substantial research tradition investigating individual factors predictive of smoking cessation, including some within the context of RCTs investigating tobacco cessation.[24,25] Our analytic plan will allow for the consideration of these individual factors alongside other predictors of tobacco cessation. There is less existing research investigating municipal-level factors related to tobacco cessation.[22] It is well known that the prevalence of smoking in adults varies across different provinces and territories of Canada (ranging from 17% in British Columbia to 63% in Nunavut).[6,8] Importantly, there are also substantial variations in smoking rates across municipal districts within these larger regions of Canada (e.g., in Ontario, rates of smoking in adults ranged from 15.4% to 44.7% between municipalities, with the highest rates generally in rural municipalities).[22,26-28] The current project will nest an RCT within a survey with linked municipal-level data. This multidisciplinary approach will allow us to determine the impact of municipal characteristics on the effectiveness of the NRT intervention.

Principal research questions

Question 1: Will an intervention of known efficacy – the distribution of free-of-cost nicotine patches sent by postal mail – have a large impact in rural regions of Canada?

Primary Hypothesis: Participants receiving the NRT package will display significantly greater quit rates (30 day abstinence) at 6-month follow-up as compared to those not offered the NRT package.

Our analysis plan also includes a test whether **participant gender moderates** the impact of the intervention.

Question 2: What other factors that systematically vary between municipalities impact the effectiveness of the nicotine patch intervention? We will use the work of Corsi et al. (2012, 2013),[22,26] as well as work by co-authors Leatherdale and Chaiton identifying geospatial factors related to smoking,[29-31] on the community-level factors related to smoking in Canada to identify relevant factors [prevalence of smoking, availability of health services, average socioeconomic status (SES), population density, region of Canada, tobacco retailer density, NRT distributor density (e.g., pharmacy, grocery store)].

While an extended set of municipal factors listed here will be collected (and examined for their predictive value as part of our secondary analyses), we focus on two contextual factors that we believe are key to why the NRT intervention may be especially impactful in rural settings.

Prevalence of Smoking Hypothesis: The impact of the NRT intervention will be larger in municipalities where there is a higher prevalence of smoking compared to municipalities where the prevalence of smoking is lower.

S***moking is more prevalent in rural regions than in urban regions***.[6-8] Because of this known higher prevalence of smoking rates in rural versus urban regions, and because the impact of the

NRT intervention appears to be unexpectedly large in rural regions, the proposed research predicts that the impact of the mailed nicotine patch intervention will be larger in regions with higher smoking rates versus those in regions with lower smoking rates. There is relevant existing research that supports this postulated differential impact. As smoking rates in an area decline, it has been suggested that those remaining in the population are more hardcore smokers (i.e., less likely to want to quit), whereas in areas with a higher smoking prevalence you are more likely to have smokers in the population who want to quit.[32-34]

Lack of Health Services Hypothesis: The impact of the NRT intervention will be larger in municipalities where there is less availability of health services compared to municipalities where there is a higher availability of health services.

Health services are more difficult to provide in areas of low population density (i.e., rural regions).[9,10] The provision of some type of help (in this case a mailed tobacco cessation aid that is well suited for a rural region) will display a larger impact in a situation where there is a lack of other services available than in one that is relatively rich in available health services.[35]

Methods

Study procedure: A two-stage recruitment process will be employed, in the context of a general population survey with a 6-month follow-up. Random digit dialling of telephone numbers from rural regions of Canada will identify households with adult (age 18 or over) smokers who smoke 10 or more cigarettes a day and who are willing to take part in a smoking study that involves two interviews. If low recruitment numbers are experienced with random digit dialling, online advertising methods (e.g. Facebook/Instagram, Kijiji volunteer section) will be used to collect telephone numbers of current adult smokers, living in rural regions who are interested in participating in a telephone survey about their smoking experiences. Should recruitment be lower than expected and the online advertising is used, interested individuals will be able to click on a link in the advertisement which will take them to a brief screener. Those found eligible (i.e. 18 years of age or older, currently smoking 10 or more cigarettes and living in an area with a rural postal code – identified using the first 3 alphanumeric Forward Sortation area code) will be asked to provide consent to be contacted by the third-party vendor contracted to complete the interviews. Individuals will be asked to provide a telephone number for this purpose (no names or other identifying information will be collected). Telephone numbers will be sent via secure file transfer to the vendor. Collection of verbal consent and baseline interview will proceed as described hereafter.

Participants will be paid $20 for the completion of each of the baseline and 6-month follow-up (payment will be made by cheque – allowing us to collect participant postal codes in order to link participant data to municipal region). Verbal consent will be obtained as the initial contact is by telephone. Only one person per household will be recruited to participate, using the method where the current smoker with the next birthday in the household is asked to participate. Both cell phone and landline numbers will be contacted. Interviews, and the remainder of the study, will be offered in both English and French. Residence of participants in rural regions will be confirmed by postal code matching. As part of the baseline survey, eligible subjects will be identified for the second recruitment – randomization of smokers into experimental and control

conditions to be offered versus not offered Nicotine Patches. A randomized half of the eligible participants will be assigned to the experimental condition and asked for their permission to have Nicotine Patches sent to their home. The 6-month follow-up survey will be conducted 6-months after the baseline survey. A letter will be sent to the participant in the 2 weeks prior to the telephone survey to notify the participant of its occurrence.

Randomization: Interviews will be conducted using computer assisted telephone interview (CATI) technology. At the end of the baseline interview, eligible subjects will be allocated to experimental and control conditions using block randomization (1:1 ratio) built into the CATI program (no stratification or minimization is necessary given the large proposed sample size in this trial).

Protecting against sources of bias: The interviewers conducting the baseline and 6-month post-intervention follow-up survey will be blind to experimental condition at the time the key outcome questions are asked. Further, while it is possible that a participant might 'unblind' themselves to an interviewer by volunteering that they used the nicotine patch, this is still unlikely to have an impact as the interviewers will be part of a separately contracted telephone survey research firm and will not be aware of the hypotheses of this research trial or be involved in the randomization process.

Inclusion criteria: The baseline telephone survey will identify adults (age 18 or over) who smoke 10 or more cigarettes a day, and who are willing to take part in a smoking study that involves two interviews. As part of the baseline survey, all participants will be asked a series of questions to assess their level of interest in receiving free NRT: "The Ministry of Health is considering different ways to help people stop smoking. One option would be to provide interested smokers with free Nicotine Patches. If Nicotine Patches were offered for free, would you be interested in receiving them?" Those who say "yes" will then be asked if they would use the Nicotine Patch to quit smoking. Those who say "yes" to this question will be asked if they would begin to use the Patch within 1 week of receiving it. A "yes" to this question will lead to being asked if they would be willing to have the Patch sent to their home. These items have been employed in our previous research trial of mailed NRT (albeit largely in urban regions).[23]
Exclusion criteria for randomized trial: Having a health condition contraindicating NRT use without the supervision of a doctor (i.e., being pregnant; having a serious heart or circulation problem, not including high blood pressure).

Interventions
Intervention group: Participants randomized to the intervention group will be asked, at the end of the baseline survey, if they want to be sent a free, 5-week supply of Nicotine Patches (example wording: "As part of a pilot trial, the Centre for Addiction and Mental Health has a supply of Nicotine Patches to distribute to interested smokers. You told us that you would be interested in receiving a free supply of Nicotine Patches. Do we have your permission to mail them directly to you at your home?"). Those agreeing will have the NRT mailed to their home the week after their baseline interview. A 5-week program of Nicotine Patches will be sent (3 weeks of Step 1 [21 mg of nicotine]; 1 week of Step 2 [14 mg of nicotine]; 1 week of Step 3 [7 mg of nicotine]). Participants will be instructed to use 1 patch per day. The mail-out will also contain a letter that advises participants to talk to their doctor if they have questions or concerns about the use of

NRT. While NRT is available over the counter, and is low-risk, should any adverse events be reported, the principal investigator will review them with an MD or pharmacist. Once randomly assigned to this condition, participants will be counted as part of the intervention group whether they agree to accept the NRT or not (intent-to-treat approach).

Control group: Participants randomly assigned to the control group will not be offered Nicotine Patches at the end of the baseline survey. An advantage of the design procedure to be employed in this trial is that participants in the control group will not have the expectation that they will receive NRT. Thus, their smoking outcomes at 6-month post-intervention follow-up will reflect a true natural history comparison to the outcomes of participants in the experimental group.

Measures

Primary Outcome Measures: The primary outcome measure will be 30-day point prevalence abstinence at 6 months post-intervention. This was also the primary outcome measure in our original trial and is a recommended outcome variable for tobacco cessation trials.[23] Continued use of the same outcome variable allows us to merge the two datasets to allow an urban comparison group for the proposed trial.

Content of baseline survey: All items have been employed by us in previous studies, including our earlier RCT of mailed NRT in a largely urban sample.[23] Participants will be asked: a) number of cigarettes smoked per day; b) level of nicotine dependence using the revised Fagerstrom test for nicotine dependence (Test-retest reliability = .78-.84);[36,37] c) number and duration of past quit attempts; d) past use of NRT and other anti-smoking medications; e) use of stop smoking services; f) intent to quit smoking in the next 6 months and 30 days; g) quantity and frequency of alcohol consumption; h) illicit drug consumption (ever and past year); and i) a series of demographic characteristics. Items assessing the inclusion criterion of interest in receiving nicotine patches will be nested within items assessing use of stop smoking medications. In addition, as smoking impacts on health-related quality of life (HRQol),[38] the HRQol will be additionally measured in all responders using the EUROHIS-QOL, an 8-item version of the World Health Organization HRQol measure.[39] Finally, questions about general health status will be asked which will include questions about health contraindications for NRT use.

Content of 6-month post-intervention follow-up survey: Participants will be asked their current smoking status, including length of time without smoking in order to assess the primary outcome variable. Participants who have not quit will be asked how many cigarettes per day they currently smoke and their intentions to quit smoking; how soon after waking they smoke their first cigarette; and if, since their baseline interview, they have stopped smoking for even one day because they were trying to quit. Participants in the experimental group will be asked if they received free NRT and, if they did, to evaluate their experiences with NRT and to elaborate on their efforts to quit smoking. These questions will include items asking whether the participants used the NRT (none, some, all) and what they did with the NRT that they did not use themselves. These questions will be asked after the core smoking status outcome measures have been assessed. Participants in the control group will also be asked about their use of NRT but the questions will be framed to reflect purchase of NRT from sources other than this ongoing study.

Participants in both conditions will also be asked about their use of other smoking cessation aids and services, including electronic cigarettes, self-help/online methods, as well as participation and/or receipt of various forms of advice and counselling from family physicians, smoking cessation specialists, or other healthcare providers. As some individuals are known to initiate use of other tobacco products after stopping use of conventional cigarettes, such as cigars or non-combustible products such as smokeless tobacco or electronic cigarettes, participants who report smoking abstinence will be asked if they currently use other tobacco or nicotine containing products. This will allow us to examine whether people who quit cigarettes compensate with other forms of nicotine delivery.

Municipal characteristics: Relevant municipal characteristics for the proposed analyses can be generated from the most recently available Canadian Community Health Survey (CCHS) data sets (average smoking rate, region, population density, average SES) and will be merged with the baseline and follow-up data collected as part of this trial. Health services availability will be generated based on publicly available data on number of MDs by location, as well as other public health services locations. The approach will be similar to that outlined by McEachern et al. (2016).[40] For the proposed trial, we will employ the database of Desktop Mapping Technologies Inc. (DMTI).[41] Enhanced Points of Interest (EPOI) is a vector GIS database of over 1 million business and recreational points of interest for all provinces/ territories in Canada (e.g., health care facilities, tobacco retailers, pharmacies). Data linkage to the participating municipalities will be consistent with linkage approaches used in previous research where geocodes for the relevant businesses are located in each municipal buffer (i.e., bounded areas for each municipality in which the different built environment characteristics are quantified). [42,43] Distance between participants' location and the closest health services available will be recorded.

Power calculations: The power calculation was conducted to estimate the sample size needed to test the primary hypothesis that participants receiving the NRT package will display significantly greater quit rates (30 day abstinence) at 6-month follow-up as compared to those not offered the NRT package. Our secondary analysis of the rural participants in our earlier trial indicated a large effect (OR = 9.59; 8.9% 30-day abstinence rate at 6 months in those receiving NRT vs 1% in those not receiving the intervention). However, this estimate is not stable due to the small sample size (n = 200 in participants from rural regions; 95% CI 1.19 to 77.16). As such, we have reduced the anticipated size of the impact of the intervention to 75% to generate the sample size estimate for the current proposal (i.e., OR = 7.1; 6.7% 30-day abstinence rate at 6 months in those receiving NRT vs 1% in those not receiving the intervention). This will allow for the likely possibility that, while the impact of the NRT intervention is large in rural regions, it may not be as large as was observed in the secondary analyses. The power analysis was based on Monte Carlo simulations. Randomly distributed dropouts were also included in the simulations, at a rate of 20%; thus accounting for the 20% attrition expected between baseline and 6-month post-intervention follow-up based on earlier trial findings.[23] Quitting smoking was the binary outcome, measured as 30-days abstinence. A sample of 290 participants per group (intervention versus control) is needed in order to obtain 80% power with 95% confidence interval. Further, analyses from our earlier trial established that 50.1% of participants from rural regions would be interested in receiving nicotine patches (and would use it within a week to quit), leading to a baseline survey needed of 1,138 participants. Finally, results from our earlier trial found that 10% of those recruited at baseline were not eligible because of reporting a health condition

contraindicating NRT use without the supervision of a doctor. Thus, in order to conduct this trial, we would need to recruit 1,252 participants for the baseline survey in order to have sufficient participants for the trial (1,138 X 110%).

Analysis Plan: Multilevel logistic modelling will be employed. The analyses are staged, with tests of the primary hypothesis (impact of the NRT intervention on 30-day abstinence rates at 6 months) as the first step and with step 2 involving tests of municipal- and individual-level factors predicted to explain the anticipated large impact of the NRT intervention in rural settings.

Step 1: Tests whether the provision of the NRT intervention impacts on 30-day abstinence rates. Experimental condition (participant randomly assigned to receive the NRT intervention package or to the control condition) is the categorical predictor in these analyses. Participants lost to follow-up will be assumed to be current smokers. As part of the step 1 analyses, we will also conduct a chi-square test to explore whether there is differential loss to follow-up between experimental conditions. Further we will replicate these analyses with all subjects with missing data excluded from the analyses. If the results are the same between the analyses where the missing data is excluded and where subjects lost to follow-up are assumed to still be current smokers, then we can safely assume that there is no differential attrition between conditions (or, at least none that impacted on the outcome of the trial). Finally, these analyses will be replicated with only those participants who provide *biochemically validation* of abstinence are coded as achieving abstinence at 6-month follow-up (all other participants are assumed to be current smokers).

Step 2: Tests to identify factors that systematically vary between individuals, and between municipalities, that explain the size of the impact of the NRT intervention on self-reported 30-day abstinence at 6-month follow-up. For these analyses, the data from urban comparison sample will be combined with the data collected in the current project of rural participants in order to allow for stronger tests. We have *pre-specified two hypothesized municipal factors* and predict that prevalence of smoking at the municipal level, and availability of health services in the municipality, will be related to the size of the impact of the NRT intervention. The multi-level logistic regression models constructed will include municipal-level sampling frame data (province or territory region of Canada), and municipal level smoking prevalence and health services availability data to determine the size of the impact of the intervention with the individual-level outcome data. Individual demographic (including participant gender) and severity of smoking characteristics will be included in order to identify individual-level factors relevant to the impact of the intervention.

Secondary analyses will add other characteristics of the municipality [average SES, population density, tobacco retailer density, NRT distributor (e.g., pharmacy, grocery store) density] to identify other contextual factors that may be important in determining the size of the impact of the NRT intervention. Additional secondary analyses will assess the effects of NRT and other predictors on relevant dependent measures pertaining to those who have not quit. These analyses will include multilevel models predicting the number of cigarettes smoked, intentions to quit, and whether participants have quit temporarily during a given time-period.

Discussion

Preventing cancer through reducing smoking rates is a cornerstone of improving the health of Canadians. Mass distributions of nicotine patches have become a major prevention initiative in Canada. The proposed trial will combine an RCT with a municipal linking strategy in order to systematically study how social and environmental factors that vary at the municipal-level will impact on the efficacy of this health intervention. What if the significant resources devoted to nicotine patch distribution where better targeted to regions where they could have the greatest impact and further, help address the health disparities associated with rural regions of Canada? This trial will allow us to answer this question, promising greater success in preventing cancer through promoting tobacco cessation.

References

1. Canadian Cancer Society/National Cancer Institute of Canada. Canadian Cancer Statistics 2007. 2007. Accessed 29 July, 2009.
2. Canadian Cancer Society's Advisory Committee on Cancer Statistics. *Canadian Cancer Statistics 2017.* Toronto, ON: Canadian Cancer Society;2017.
3. U.S. Department of Health and Human Services (USDHHS). *The Health Consequences of Smoking - 50 Years of Progress.* Rockville, MD2014.
4. Health Canada. Canadian Tobacco, Alchol an Drugs Survey (CTADS), summary of results for 2017. 2018; https://www.canada.ca/en/health-canada/services/canadian-tobacco-alcohol-drugs-survey/2017-summary.html#n2. Accessed November 15, 2018, 2017.
5. U.S. Department of Health and Human Services (USDHHS). *The health benefits of smoking cessation.* 1990. DHHS Publication (CDC) 90-8416.
6. Statistics Canada. *The Daily — Canadian Community Health Survey, 2014.* 2015.
7. Cunningham JA, Kushnir V. Effectiveness of mass distribution of nicotine patches to promote tobacco control in rural versus urban settings. *J Epidemiol Community Health.* 2017;71(5):519.
8. Ministerial Advisory Council on Rural Health. *Rural health in rural hands: Strategic directions for rural, remote, northern and aboriginal communities.* Health Canada;2002.
9. Standing Council on Health. *National Strategic Framework for Rural and Remote Health.* 2012.
10. College of Family Physicians of Canada (CFPC). *The Rural Rob Map for Action - Directions.* Mississauga, On2017.
11. Kondro W. Health of rural Canadians lags behind urban counterparts. *CMAJ.* 2006;175(10):1195.
12. Deering KN, Lix LM, Bruce S, Young TK. Chronic Diseases and Risk Factors in Canada's Northern Populations: Longitudinal and Geographic Comparisons. *Can J Public Health.* 2009;100(1):14-17.
13. Stevenson CE, Mannan H, Peeters A, et al. The effect of modifiable risk factors on geographic mortality differentials: a modelling study. *Bmc Public Health.* 2012;12.
14. Stead LF, Perera R, Bullen C, et al. Nicotine replacement therapy for smoking cessation. *Cochrane Database Syst Rev.* 2012;11:CD000146.
15. Hartmann-Boyce J, Chepkin SC, Ye WY, Bullen C, Lancaster T. Nicotine replacement therapy versus control for smoking cessation (Review). *Cochrane Db Syst Rev.* 2018(5).
16. Cummings KM, Fix B, Celestino P, Carlin-Menter S, O'Connor R, Hyland A. Reach, efficacy, and cost-effectiveness of free nicotine medication giveaway programs. *J Public Health Manag Pract.* 2006;12(1):37-43.
17. Miller N, Frieden TR, Liu SY, et al. Effectiveness of a large-scale distribution programme of free nicotine patches: a prospective evaluation. *Lancet.* 2005;365(9474):1849-1854.
18. Davis KA, Coady MH, Mbamalu IG, Sacks R, Kilgore EA. Lessons learned from the implementation of a time-limited, large-scale nicotine replacement therapy giveaway program in new york city. *Health Promot Pract.* 2013;14(5):767-776.
19. Selby P, Zawertailo L, Dragonetti R. *The STOP (STOP Smoking Treatment for Ontario Patients) Study. 6-month effectiveness of mass distribution of NRT in Ontario.* 2006.

20. Zawertailo L, Dragonetti R, Bondy SJ, Victor JC, Selby P. Reach and effectiveness of mailed nicotine replacement therapy for smokers: 6-month outcomes in a naturalistic exploratory study. *Tob Control.* 2013;22(3):e4.
21. Borland R, Pigott R, Rintoul D, Shore S, Young S. *Barriers to access of smoking cessation programs, nicotine replacement therapy and other pharmacotherapies for the general Australian population and at-risk population groups.* Canberra: VicHealth Centre for Tobacco Control, Cancer Council Victoria;2002.
22. Corsi DJ, Lear SA, Chow CK, Subramanian SV, Boyle MH, Teo KK. Socioeconomic and geographic patterning of smoking behaviour in Canada: a cross-sectional multilevel analysis. *PLoS ONE.* 2013;8(2):e57646.
23. Cunningham JA, Kushnir V, Selby P, Tyndale R, Zawertailo L, Leatherdale S. Mailing nicotine patches to promote tobacco cessation among adult smokers: Primary outcomes of a randomized clinical trial. *JAMA Intern Med.* 2016;176(2):184.
24. Kushnir V, Sproule BA, Cunningham JA. Mailed distribution of free nicotine patches without behavioral support: Predictors of use and cessation. *Addict Behav.* 2017;67:73-78.
25. Wetter DW, Kenford SL, Smith SS, Fiore MC, Jorenby DE, Baker TB. Gender differences in smoking cessation. *J Consult Clin Psych.* 1999;67(4):555-562.
26. Corsi DJ, Chow CK, Lear SA, Subramanian SV, Teo KK, Boyle MH. Smoking in context: a multilevel analysis of 49,088 communities in Canada. *Am J Prev Med.* 2012;43(6):601-610.
27. Corsi DJ, Boyle MH, Lear SA, Chow CK, Teo KK, Subramanian SV. Trends in smoking in Canada from 1950 to 2011: progression of the tobacco epidemic according to socioeconomic status and geography. *Cancer Causes Control.* 2014;25(1):45-57.
28. Meng G, Brown KS, Thompson ME. Spatial and temporal patterns of smoking prevalence in Ontario. *BMC Public Health.* 2015;15:182.
29. Chaiton MO, Mecredy G, Cohen J. Tobacco retail availability and risk of relapse among smokers who make a quit attempt: a population-based cohort study. *Tob Control.* 2017.
30. Chan WC, Leatherdale ST. Tobacco retailer density surrounding schools and youth smoking behaviour: a multi-level analysis. *Tob Induc Dis.* 2011;9.
31. Leatherdale ST, Strath JM. Tobacco retailer density surrounding schools and cigarette access behaviors among underage smoking students. *Ann Behav Med.* 2007;33(1):105-111.
32. Costa ML, Cohen JE, Chaiton MO, Ip D, McDonald P, Ferrence R. "Hardcore" definitions and their application to a population-based sample of smokers. *Nicotine Tob Res.* 2010;12(8):860-864.
33. Darville A, Hahn EJ. Hardcore smokers: what do we know? *Addict Behav.* 2014;39(12):1706-1712.
34. Ip DT, Cohen JE, Bondy SJ, et al. Do components of current 'hardcore smoker' definitions predict quitting behaviour? *Addiction.* 2012;107(2):434-440.
35. Rose G. *The strategy of preventive medicine.* Oxford: Oxford University Press; 1992.
36. Heatherton TF, Kozlowski L, Frecker RC. The Fagerstrom Test for Nicotine Dependence: A revision of the Fagerstrom Tolerance Questionnaire. *B J Addict.* 1991;86:1119-1127.

37. Pomerleau CS, Carton SM, Lutzke ML, Flessland KA, Pomerleau OF. Reliability of the Fagerstrom Tolerance Questionnaire and the Fagerstrom Test for Nicotine Dependence. *Addict Behav.* 1994;19(1):33-39.
38. Sales MP, Oliveira MI, Mattos IM, Viana CM, Pereira ED. The impact of smoking cessation on patient quality of life. *J Bras Pneumol.* 2009;35(5):436-441.
39. Schmidt S, Muhlan H, Power M. The EUROHIS-QOL 8-item index: psychometric results of a cross-cultural field study. *Eur J Public Health.* 2006;16(4):420-428.
40. McEachern J, Ahamad K, Nolan S, Mead A, Wood E, Klimas J. A Needs Assessment of the Number of Comprehensive Addiction Care Physicians Required in a Canadian Setting. *J Addict Med.* 2016;10(4):255-261.
41. DMTI Spatial Inc. Canmap Route Logistics (CMRL) & Enhanced Points of Interest (EOPI). 2017.
42. Leatherdale ST, Pouliou T, Church D, Hobin E. The association between overweight and opportunity structures in the built environment: a multi-level analysis among elementary school youth in the PLAY-ON study. *Int J Public Health.* 2011;56(3):237-246.
43. Pouliou T, Elliott SJ. Individual and socio-environmental determinants of overweight and obesity in Urban Canada. *Health Place.* 2010;16(2):389-398.
