# Supplementary material for: Effect of Postal-Mailed Nicotine Patches on Tobacco Cessation Among Smokers in Rural Canada: A Randomized Clinical Trial
Source: JAMA Netw Open. 2023 Jul 24;6(7):e2325206. doi: 10.1001/jamanetworkopen.2023.25206 (PMC10366697; doi:10.1001/jamanetworkopen.2023.25206)
Supplement: Supplement 2. — Data Sharing Statement [file jamanetwopen-e2325206-s002.pdf]

Data Sharing Statement

Cunningham. Effect of Postal-Mailed Nicotine Patches on Tobacco Cessation Among Smokers in Rural Canada—A Randomized Clinical Trial. *JAMA Netw Open*. Published July 24, 2023. doi:10.1001/jamanetworkopen.2023.25206

Data
Data available: No

Additional Information
Explanation for why data not available: Available from the first author upon reasonable request.
